# Rhinosinusitis derived Staphylococcal enterotoxin B possibly associates with pathogenesis of ulcerative colitis

**DOI:** 10.1186/1471-230X-5-28

**Published:** 2005-09-06

**Authors:** Ping-Chang Yang, Tao Liu, Bin-Quan Wang, Tao-Yuan Zhang, Zi-Yuan An, Peng-Yuan Zheng, Dao-Fa Tian

**Affiliations:** 1 Department of Pathology and Molecular Medicine, McMaster University, Hamilton, ON, Canada; 2 Department of Otolaryngology, Shanxi Medical University, the First Hospital, Taiyuan, China; 3 Department of Gastroenterology, Shanxi Medical University, the First Hospital, Taiyuan, China; 4 Department of Gastroenterology, Zhengzhou University, the Second Hospital, Zhengzhou, China; 5 Department of Otolaryngology, Hunan University of Chinese Traditional Medicine, Changsha, China; 6 an adjunct Professor of Allergy Unit and Department of Otolaryngology, Shanxi Medical University, Taiyuan, China

## Abstract

**Background:**

During clinical practice, we noticed that some patients with both ulcerative colitis (UC) and chronic rhinosinusitis (CRS) showed amelioration of UC after treatment of CRS. This study was designed to identify a possible association between CRS and UC.

**Methods:**

Thirty-two patients with both CRS and UC received treatment with functional endoscopic sinus surgery (FESS) for CRS. Clinical symptom scores for CRS and UC, as well as serum levels of anti-Staphylococcal enterotoxin B (SEB) were evaluated at week 0 and week 12. Sinus wash fluid SEB content was measured with enzyme-linked immunosorbent assay (ELISA). The surgically removed tissues were cultured to identify growth of *Staphylococcus. aureus *(*S. aureus*). Immunohistochemistry was employed to identify anti-SEB positive cells in the colonic mucosa. Colonic biopsies were obtained and incubated with SEB. Mast cell activation in the colonic mucosa in response to incubation with SEB was observed with electron microscopy and immunoassay.

**Results:**

The clinical symptom scores of CRS and UC severe scores (UCSS) were significantly reduced in the UC-CRS patients after FESS. The number of cultured *S. aureus *colonies from the surgically removed sinus mucosa significantly correlated with the decrease in UCSS. High levels of SEB were detected in the sinus wash fluids of the patients with UC-CRS. Histamine and tryptase release was significantly higher in the culture supernate in the patients with UC-CRS than the patients with UC-only and normal controls. Anti-SEB positive cells were located in the colonic mucosa.

**Conclusion:**

The pathogenesis of UC in some patients may be associated with their pre-existing CRS by a mechanism of swallowing sinusitis-derived SEB. We speculate that SEB initiates inappropriate immune reactions and inflammation in the colonic mucosa that further progresses to UC.

## Background

Ulcerative colitis (UC) is a relapsing chronic inflammatory disease of the colon with unknown etiology. The evidence from animal models suggests that an altered immune response towards the commensal gut microbiota plays a key role in the development and perpetuation of this pathological condition [1,2]. Treatment of UC is still a hot topic in clinical research. Although therapeutics has been advanced greatly in the recent years [3-5], generally inflammatory suppression with glucocorticoids and/or sulfasalazine is still the primary therapy for UC [6,7]. However, a large number of patients are resistant or intolerant to medical treatment [8].

The accumulation of evidence from animal studies, cell culture studies, and clinical observations has indicated an important role for microbes and/or their products in the induction or exacerbation of enterocolitis [9]. Microbial antigen Staphylacoccal enterotoxin B (SEB) has been shown to induce enteropathy, characterized by altered villus-crypt in BALB/c and T cell-reconstitued SCID mice [10]. Oral feeding of SEB leads the development of colitis in the absence of regulatory T cells [11]. SEB has been shown to cross the intestinal epithelial cell barrier [12] and is implicated in inducing inappropriate immune reactions in humans [13] and mice [14] leading to activation of T cells to produce proinflammatory cytokines such as IL-1, IL-2, TNF-α and IFN-γ [15,16]. SEB is capable of inducing inflammatory cytokines release from explants of colonic biopsies from patients with IBD [17]. However, our knowledge about the association between SEB and IBD is still limited. SEB is synthesized as a precursor protein of 266 amino acids. This precursor is then activated during excretion by cleavage of the N-terminal portion of the molecule. The active enterotoxin B is a single 239 amino acid chain of molecular weight 28,000 Daltons and isoelectric point of 8.6 [18]. SEB is a superantigen and possesses powerful immune regulatory capabilities that result in increased T cell activation and proliferation. SEB-treated Balb/c mice display a dose-dependent colonic inflammation [19]. SEB can also induce colonic epithelial barrier dysfunction [20] that promotes uptake of exogenous antigens [21], microbial products and other noxious substances into the intestinal tissue to contact immune cells and to initiate immune reactions.

Over the past 15 years, more than 2000 patients with chronic rhinosinusitis (CRS) including some patients with both CRS and UC (UC-CRS) visited our clinic. Their CRS was treated with different remedies, including medical treatment and functional sinus endoscopic surgery (FESS). Apart from the satisfactory efficacy in the treatment of CRS, some of the patients with UC-CRS showed great amelioration of UC as well that couldn't be explained with the primary treatment alone. Therefore, we postulated that there might be an association between CRS and UC in these patients. Microbial infection is the most common cause of CRS. The microbial products produced in the sinuses, such as SEB [22,23] can be discharged into the nasal cavity with secretions through the natural ostia going backward to the pharynx, and then swallowed. After reaching the gastrointestinal tract, SEB is capable of affecting intestinal mucosal physiological functions [24,25]. Therefore, this prospective study was designed to identify a possible association between CRS and UC.

The nasal cavity and sinus are primary sites of colonization by *S. aureus *[26], and SEB was detected in the nasal cavity of the patients with CRS, nasal polyposis and allergic rhinitis [22,23,27,28]. Based on clinical observations, we hypothesized that sinusitis-derived SEB played a role in the pathogenesis of chronic inflammation in the intestinal mucosa via impairing the epithelial barrier function and inducing inappropriate immune reactions. In this study, we aimed to investigate if (i) colonization of *S. aureus *can be identified in the sinus cavity of the patients with UC-CRS; (ii) SEB production can be detected in the sinuses; (iii) the improvement of UC associates with the removal of inflammation from the sinuses; (iv) immune cells in the colonic mucosa have been and can be activated by SEB.

## Methods

In this study, we evaluated the effect of FESS in the treatment of a group of patients with refractory CRS and moderate UC. The study protocol was approved by the research ethics committee at the First Hospital, Shanxi Medical University. All patients gave written informed consent prior to recruitment. Surgical intervention was chosen for the patients because they had tried different therapies that failed to cure their CRS. The clinical condition of UC was moderate and suitable for sinus surgery. Those patients with severe UC were excluded. Unless otherwise mentioned, all the reagents used in this study were purchased from Sigma Aldrich (USA).

Thirty two patients with refractory CRS and moderate UC were enrolled in this study. Another 32 patients with moderate UC but no CRS were selected as a control group. Twenty-five healthy medical students were enrolled in this study as a healthy control group, their serum and nasal wash fluids were collected as controls. We diagnosed CRS as inflammation of the sinus mucosa with a persistent mucoid or mucopurulent nasal discharge for longer than 3 months that resisted antimicrobial therapy and antral irrigation. Diagnosis was made on the basis of clinical history, rhinoscopic findings, and computed tomographic scan of rhinosinuses. CRS was confirmed by computed tomographic examination that showed diffuse mucosal thickening in the ethmoid or/and maxillary sinuses bilaterally with scores higher than 12 by the Lund-Mackay staging system [29].

The diagnosis and treatment of UC was carried out by one gastroenterologist (Dr An Z). The diagnosis of UC [30] was based on clinical history, colonoscopy and histology. The history included persistent bloody diarrhea, rectal urgency, or tenesmus, stool. Examinations, sigmoidoscopy and biopsy were performed to confirm the presence of colitis and to exclude the presence of infectious etiologies. UC severity scores (UCSS) [31] were recorded for each patient on admission (week 0) and week 12. The UCSS were evaluated from 4 clinical items: stool frequency, rectal bleeding, sigmoidoscopic appearance, and the physician's global assessment.

### Sinus and nasal wash fluids collection

Every patient with UC-CRS underwent maxillary sinus puncturing and irrigating with saline. The SWF was collected prior to other procedures. Five ml saline was injected into the sinus cavity and recollected and stored at -70°C for further analysis. Protein content in SWF was evaluated with UV spectrophotometer (Beckman DU-65) at 280 nm. Contents of SEB, SEA and TSST1 in SWF were evaluated with ELISA. Nasal lavage fluids (5 ml saline) instead of sinus irrigation were collected from the healthy controls and UC controls. None of the subjects had acute upper respiratory acute infections within the past month.

### Treatment

The procedures of FESS followed the previous reports [32]. Penicillin G (800,000 IU, i.m., Bid) was administered after the surgery for 3 days (no patient in this group showed allergic signs for penicillin G). The surgical side of sinus was irrigated daily until the irrigated fluids became clear. After discharge, the patients were required to visit the hospital to conduct a post-operation examination of nasal cavity and sinuses on a monthly basis for the first year. Any unusual feelings in the operated sinus area were recorded. Physicians would do necessary treatment for relapse of inflammation in the sinuses during the entire follow-up period. The treatment of UC including sulfasalazine and corticosteroids continued for each patient during the 12-week observation period including the patients with UC-CRS and the patients with UC.

### Serology of C-reaction protein, IgE and cytokines

Blood samples were collected at week 0 and week 12. The serum was separated and analyzed for contents of C reactive protein and SEB specific IgE respectively. The methods were done as previously reported. [33,34] Blood samples were also collected from the healthy controls and UC-controls twice, 12 weeks apart.

### *S. aureus *identification

Surgically removed tissues were obtained from each patient (a nasal lateral wall swab was obtained from UC-only patients and healthy controls instead) and were sent to the Microbiological Laboratory within 30 minutes. 100 mg mucosa was homogenized in 0.1 ml saline. The same volume of the homogenates from each patient was cultured aerobically on blood agar plates. Colonies of different morphology growing on the agar were separately enumerated, subcultured on blood agar, Gram stained, and tested for catalase production. Isolates with a typical Gram-stained appearance and a positive catalase test were tested for coagulase production, and positive isolates were regarded as *S. aureus*. The template DNA was extracted from the positive cultured colony of each subject with the procedures reported by Riffon [35]. PCR was performed with the samples from each subject. The nucleotide sequence is available at the GenBank data library (accession number, CP000046). The primers were designed using the software Primer3 and the specificity was determined with Blast. The primers were: 5'-ttgcatatccgcgtcaaata-3' and 5'-cttcatgttctttcgcatcg-3'. Amplification was performed on a Perkin-Elmer (Norwalk, Conn.) thermocycler in 25-μl reaction mixtures. The program consisted of an initial denaturation step at 94°C for 2 min, followed by 22 cycles of 1 s at 94°C, 2 s at 58°C, and 10 s at 72°C, with a final extension step at 72°C for 5 min. Amplification products were separated on a 1% agarose gel and stained with ethidium bromide before being analyzed on a UV bench by using GelDoc2000 (Bio-Rad). The PCR products were routinely sequenced to confirm amplification of the targeted sequence.

### Immunohistochemistry

Two pieces of colonic tissue at the edge of ulcers was taken during colonoscopy from each patient. One biopsy was snap frozen with liquid nitrogen in Tissue Tek. Cryosections were air dried overnight and fixed with cold acetone for 15 minutes. The endogenous peroxidase was quenched by incubation in 0.3% H_2_O_2 _for 30 minutes and non-specific binding was blocked by incubating the sections in 10% goat serum/phosphate-buffered saline. Specimens were incubated with mouse anti-SEB monoclonal antibody or IgG isotype control immunoglobulin. Subsequently, after incubation with secondary rabbit anti-mouse antibody (HRP conjugated, Vector Laboratories), color was developed applying the Vectastain peroxidase detection kit and diaminobenzidine as a substrate. Counterstaining was performed with hematoxylin. The negative control slides omitted the first antibody or added the isotype control instead.

### Tryptase and histamine release from the cultured colonic biopsies in response to the stimulation of SEB

One of the two intestinal biopsy specimens from each patient was subjected to histamine and tryptase release evaluation in response to SEB stimulation. Briefly, upon removal, the specimens were weighed in a sterile vial containing 1 ml pre-warmed (37°C) and oxygenized RPMI 1640 culture medium (no serum). The specimen containing vials were incubated for 30 minutes at 37°C in a humidified 95% and air containing 5% CO_2 _atmosphere and environment and rocked gently at 20 cycles/min. The supernatants were carefully collected and temporally kept at 4°C. The specimens were washed with pre-warmed no-serum RPMI medium for 3 times, then another RPMI 1640 medium (no serum) with 20 μl SEB/ml was added to the vial and incubated for another 30 minutes. The colonic tissues were carefully taken out and immersed to 2% glutaraldehyde immediately. The supernatants were centrifuged at 17,530 × g for 10 minutes at 4°C. The supernatants were stored at -70°C for further assay. Eight mucosal specimens from surgical removed colonic tissue of the patients with colonic cancer were collected. Samples were selected from the uninvolved part and processed as normal controls.

For tryptase activity assay, triplicate aliquots (10 μL) of the supernatants were added to 200 μL of buffer (50 mmol/L Tris/HCl, pH 7.6, 120 mmol/L NaCl, 20 μg/mL heparin) containing 0.5 mmol/L substrate (tosyl-Glycine-Proline-Arginine-pNitroanilide) and incubated at room temperature for 17 hours (± inhibitors as indicated). Cleavage of the substrate was measured using a microtiter plate reader (absorbance 405 nm) and normalized to the weight of the biopsy specimens used in each specimen.

For histamine assay, triplicate aliquots (50 μL) of the supernatants using a selective enzyme-linked immunoassay (Immunotech, Marseille, France) according to the manufacturer's directions. Histamine was measured using a microtiter plate reader (absorbance 405 nm) and normalized to the weight of the biopsy specimens.

### Electron microscopy

After incubation with SEB, the colonic biopsies were fixed with 2% glutaraldehyde for 2 hours at room temperature and postfixed in 1% osmium tetroxide for 1 hour. After dehydration and embedding in EPON, ultra thin section were cut and collected on 200 mesh grids, stained with uranyl acetate and lead citrate, and observed with a JEC 1200 transmission electron microscope. Thirty mast cells were selected randomly from each specimen and photographed. The granules of the selected mast cells were categorized into three types: intact, piecemeal degranulation (PMD) and anaphylactic degranulation (AND) according to reported criteria [36].

### Statistical analysis

Data were expressed as means ± standard deviation (SD). Differences between groups were analyzed with the *student t *test or χ^2 ^test. Correlationship between groups was demonstrated by correlation analyses. The significant criteria was set at p < 0.05.

## Results

### Clinical symptoms of CRS improved after FESS

**Figure 1:**
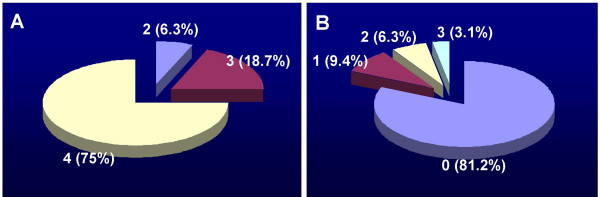
Chronic rhinosinusitis clinical scores of the patients with both chronic rhinosinusitis (CRS) and ulcerative colitis (UC) was recorded before (week 0, A) and after (week 12, B) FESS. 0 stands no symptom; 4 stands severe symptom. The numbers stand for clinical scores (percentage of patient). The results of χ^2 ^analyses showed p < 0.05 as compared the clinical scores of week 0 with that of week 12.

All the CRS patients underwent FESS in their maxillary sinusitis. Twelve (37.5%) patients also underwent bilateral ethmoidectomy. Eight patients also underwent unilateral ethmoidectory (25%, right or left side). Four (12.5%) patients also underwent septum orthomorphia. Fourteen (43.75%) patients had nasal polyps that were removed during FESS. Five (15.63%) patients underwent partial middle turbinectomy. Six (18.75%) patients underwent inferior turbinectomy. During the 12-week observation period, all the patients underwent regular follow-up visit (The total follow-up period lasted for one year after FESS). Twelve weeks after FESS, all the patients reported significant improvement in their clinical symptoms of CRS. Clinical symptom scores were recorded at week 0 and week 12 that were defined from 0 to 4. 0 for no symptoms; 4 for severe. Most patients had moderate to severe CRS clinical symptoms before FESS; 29 out of 32 (90.62%, scored 0 and 1) patients showed significant reduction of their clinical scores of sinusitis; 21 out of 32 (65.63%) scored 0 and 1 (Fig 1).

### Effect of treatment with FESS on UC clinical symptoms

**Figure 2:**
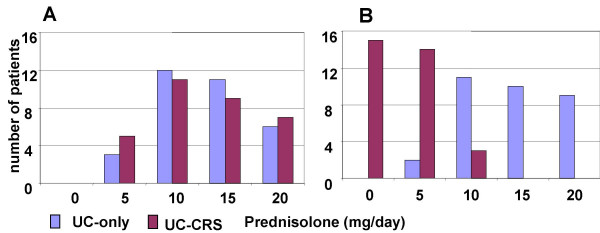
Prednisolone requirement was recorded from the patients with UC-CRS and the patients with UC at week 0 (A) and week 12 (B). The bars represent the number of patients. χ^2 ^analyses were performed with the data between the two groups. The results showed p > 0.05 at week 0 and p < 0.05 at week 12.

**Figure 3:**
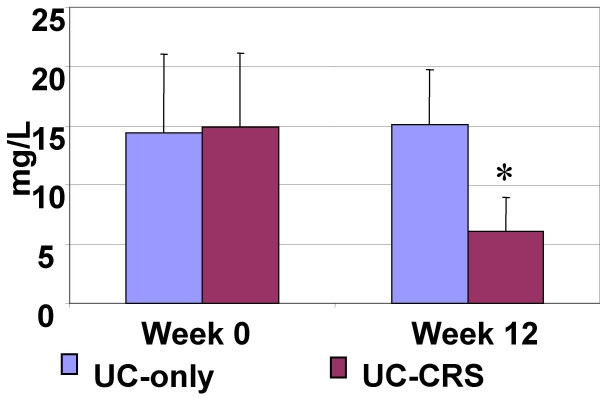
Serum C reactive protein. Bars stand for serum levels of C reactive protein. Data were presented mean ± SD. t test was performed with the data before and after FESS. *, p < 0.05, compared with UC-only group. UC-only: patients with UC only. UC-CRS: patients with both UC and CRS.

The demographic characteristics of the patients in the present study are listed in Table 1. In the period of 12 week observation period, treatment for UC of the patients in this study were carried out by one gastroenterologist according to routine therapy. After FESS, the requirement for steroids was gradually reduced in the UC-CRS group and was significantly less than the UC group (Fig 2). From week 0 to week 12, levels of C reactive protein rose in control patients (with UC only) who received routine treatment rather than FESS, from 4 to 20 mg/l, whereas the value reduced significantly in the FESS group. The C reactive protein in the healthy control group was under detectable levels (Fig 3).

**Figure 4:**
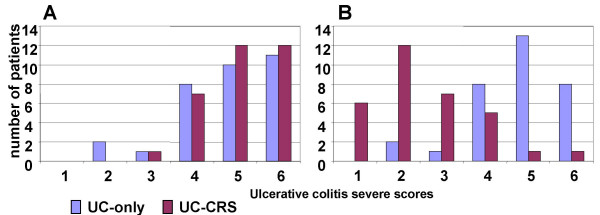
Ulcerative colitis severe scores of the patients with UC-CRS and the patients with UC-only were recorded at week 0 (A) and week 12 (B). Bars represent summary scores from clinical observation. Data were presented as numbers of patients of each score. χ^2 ^analyses were performed with the data between the two groups. The results showed p > 0.05 at week 0 and p < 0.05 at week 12.

Sigmoidoscopy was performed in all subjects during admission and 12 weeks after FESS. The control UC patients were also examined with sigmoidoscopy in the same period. The results showed that the patients with both UC-CRS significantly improved in appearance of the colonic mucosa after FESS, while UC patients showed no change. UCSS assessment also showed significant improvement in the patients with UC-CRS after FESS. The UCSS did not show much difference in the patients with UC-only although they received the routine treatment for their UC (Fig 4).

### *S. aureus *was identified in the sinuses of the patients with CRS

**Figure 5:**
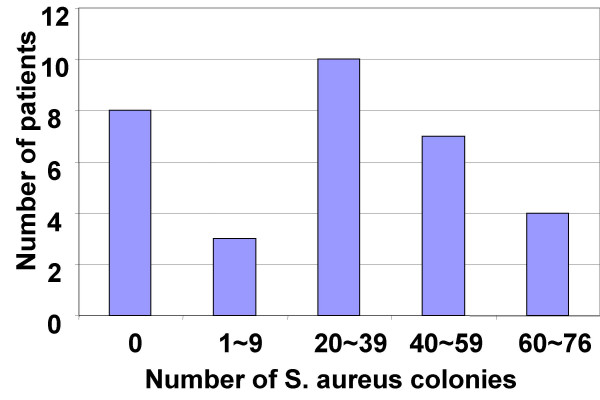
S. aureus was cultured with the surgical removed sinus mucosa of the patients with UC-CRS. The method using to identify S. aureus was described in the text. Bars stand for numbers of patients.

**Figure 6:**
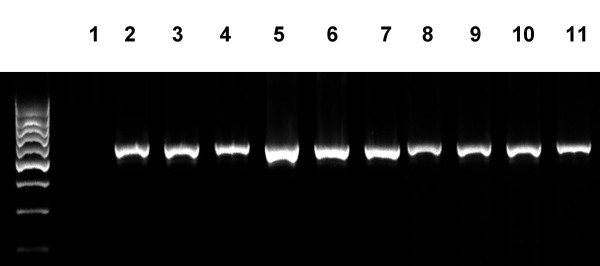
RT-PCR results of *S. aureus *DNA amplification. DNA templates were extracted from the surgical removed sinus tissue of the patients with UC-CRS. Lane 1 is negative control by adding water instead of DNA template. Lanes 2–11 are representative PCR bands of *S. aureus *DNA amplification.

**Figure 7:**
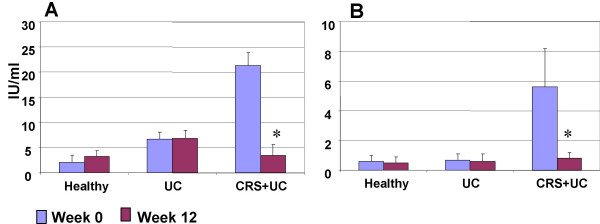
Serum IgE antibody. Serum samples were collected from all the subjects at week 0 and week 12. IgE levels were evaluated with ELISA. Data were presented as mean ± SD. *, p < 0.05, compared with the same group at week 0. A, serum total IgE; B, serum anti-SEB IgE.

Bacterial culture results showed *S. aureus *growth in the surgically removed tissue in 24 (75%) patients with UC-CRS. The numbers of S. aureus colonies in the culture ranged from 0 to 76 with an average of 28.5/patient. The reduction of UC clinical symptom scores significantly correlated with the number of the cultured *S. aureus *colonies from the surgical removed sinus mucosa (Fig 5, r = 0.8399, p < 0.0001). PCR results showed *S. aureus *DNA amplified products from the surgical removed tissue of 32 (100%) patients that further confirmed the existence of *S. aureus *infection in the sinuses of patients with UC-CRS (Fig 6). RT-PCR amplified product sequence analysis confirmed that the amplified bands were consistent with the target DNA sequences. Fig 7 depictes serum IgE assay results. Serum specific IgE and total IgE levels were evaluated with ELISA. Before FESS, serum anti-SEB antibody levels were significantly higher in the patients with UC-CRS than the patients with UC only and the healthy controls (p < 0.05). The levels of specific IgE in UC-CRS patients were significantly decreased as measured at week 12 after FESS (p < 0.05). The levels of total IgE were also higher in UC-CRS patients before FESS compared with UC-only patients and healthy controls (p < 0.05). The results showed the total IgE levels were also significantly decreased after FESS as compared with the results before FESS (p < 0.05). A ratio of specific IgE/total IgE was calculated and listed as: control group (28.6% and 15.6%; week 0 and week 12); UC-only group (10.6% and 8.8%) and UC-CRS group (26.3% and 23.5%). Ratio statistical analysis showed that differences between week 0 and week 12 didn't reach significance (p > 0.05) in all three groups indicating the same tendency of change in total IgE and the SEB specific IgE.

### SEB specific antibody positive stained cells were located in the colonic mucosa. High concentration of SEB was evaluated in the sinus wash fluids

**Figure 8:**
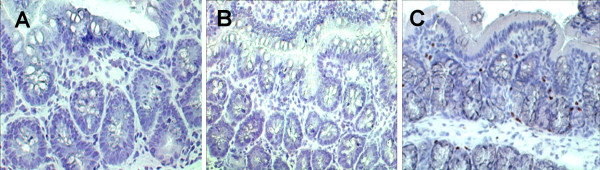
Anti-SEB positive stained cells were located in the colonic mucosa. Colonic biopsies were got from the patients with UC-only (A) and the patients with UC-CRS (B and C). Cryosections were stained with anti-SEB antibody or IgG isotype control immunoglobulin (B). Sections were visualized by the immunoperoxidase method and counterstained with hematoxylin. SEB specific IgE-bearing cells were stained in brown (C). ×200.

**Figure 9:**
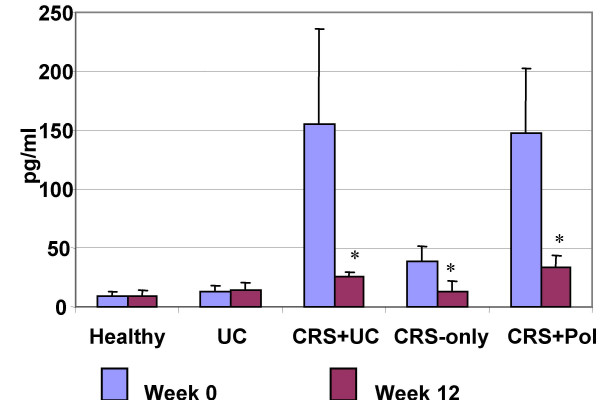
SEB content in sinus wash fluids (nasal lavage fluids for the groups of UC-only and healthy controls). Bars stand for SEB content. Data were presented as mean ± SD. *, p < 0.05, compared with the same group at week 0. CRS-only: a group of 12 patients with CRS but not UC. CRS-Pol: a group of 12 patients with CRS and nasal polyposis.

Immunohistochemistry showed anti-SEB positive staining cells in the colonic mucosa of patients with UC-CRS, but not in patients with UC only (Fig 8). Sinus wash fluids were evaluated for SEB content. A significantly higher SEB level was found in the sinus wash fluids from patients with UC-CRS. SEB content was much lower in the nasal wash fluids of the UC-only patients and healthy controls. Sinus wash fluids were also colleted from 12 patients with CRS, but without UC and 12 patients with nasal polyposis and CRS, but without UC at week 0 and week 12 after FESS. SEB content in the sinus wash fluids was also evaluated (Fig 9). The contents of SEA and TSST1 in the sinus wash fluids were undetectable.

### Colonic mucosal mast cell degranulation in response to SEB stimulation ex vivo

**Figure 10:**
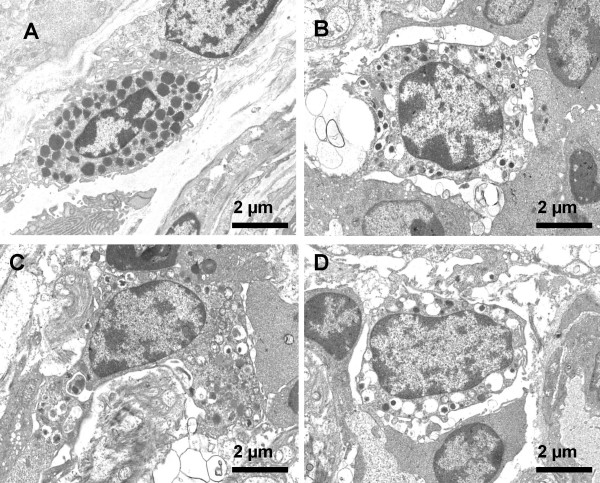
Mast cells in the colonic mucosa. Representative electron photomicrographs of mast cells were taken from the colonic mucosal specimens of A: normal controls, B: patients with UC-only and C and D: patients with UC-CRS (C, before stimulated with SEB; D, after stimulated with SEB). ×3,000.

**Figure 11:**
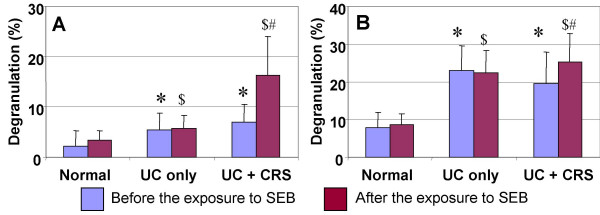
The ratio of mast cell degranulation in the colonic mucosa. Under an electron microscope, mast cell degranulation was categorized into piecemeal degranulation (PMD) and anaphylactic degranulation (AND). Percentage of degranulation was calculated with the number of total granules and the number of PMD or AND. *Student t *test was performed with the data between groups. *, P < 0.05, compared with PMD of normal group (A). #, p < 0.05, compared with PMD of normal group (B). $, p < 0.05, compared with AND of the normal group (A). &, p < 0.05, compared with AND of the normal group (B). @, p < 0.05, compared with PMD of UC only group (A). +, p < 0.05, compared with AND of UC only group (B).

The numbers of mast cells in the colonic mucosa were counted with light microscopy. There were significantly more mast cells in patients with UC-only and UC-CRS compared with normal control colonic mucosa. As depicted in Figures 10 and 11, electron microscopy revealed three types of granules in the mast cells of the colonic mucosa, intact, PMD and AND. In the normal colonic mucosa, there was a low ratio of degranulation in the colonic mast cells (PMD, 7.7 ± 4.2%; AND, 2.2 ± 3.1%). A significant increase in the ratio of degranulation of both PMD and AND was observed in colonic specimens of patients with UC-only and UC-CRS before SEB stimulation. A great difference was observed in the mast cell degranulation in the colonic mucosa in response to SEB stimulation between the groups with UC only and UC-CRS ex vivo. AND type degranulation was significantly increased in patients with UC-CRS compared to UC only patients (p < 0.05).

### Tryptase and histamine release in response to SEB stimulation ex vivo

**Figure 12:**
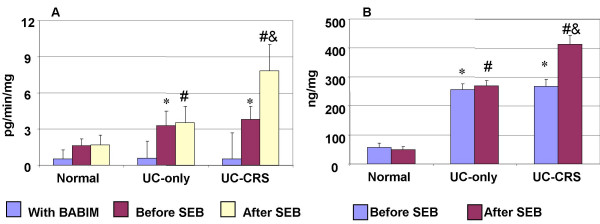
Tryptase and histamine release from the colonic mucosal biopsy specimens in healthy controls, patients with UC-only and patients with UC-CRS. Bars represent contents of tryptase (A) or histamine (B) in the supernatant of the culture of the colonic biopsies. * and #, p < 0.05, compared with the normal control samples; $, p < 0.05, compared with UC-only patients.

Tryptase and histamine was detected in the culture of the colonic biopsies in the first 30-minute. The levels of tryptase and histamine were approximately 3 times higher in the specimens from patients with UC or UC-CRS compared to normal controls. The release of tryptase and histamine further increased after incubation with SEB in the UC-CRS group, but not in the UC group. The tryptase inhibitor BABIM significantly inhibited the effect of tryptase (Fig 12).

## Discussion

We have observed the clinical outcome of a group of patients with both CRS and UC after treatment with FESS. Clinical symptom scores of both CRS and UC were significantly reduced 12 weeks after FESS. UC-CRS patients exhibited sinus infection with *S. aureus *high levels of anti-SEB antibody in the serum, and anti-SEB antibody positive stained cells in the colonic mucosa. In particular, the mast cells in the colonic biopsy specimens from the patients with UC-CRS were activated by incubation with SEB by showing extensive degranulation and release of histamine and tryptase. This is consistent with Dionne's study [17] that reported SEB induced IBD rectal biopsies to release inflammatory mediators ex vivo such as TNF-α and IL-1.

During the 12-week observation period, both groups of UC patients with or without CRS were treated with the routine remedies for UC, mainly sulfasalazine and glucosteroids. Therefore, the marked improvement of UC in patients with UC-CRS should be the removal of inflammation in the sinuses by FESS. Although brief (3 days) penicillin G administration was given to patients after FESS, it did not likely contribute to the amelioration of UC, because penicillin G mainly effects on acute Gram positive bacterial infection which grow fast and have high requirement of cell wall synthesis whereas UC is a chronic inflammatory disease. The prevalence of Crohn's disease, another form of IBD, was elevated in patients with chronic sinonasal disease, occurring in approximately one-half of patients followed at a tertiary IBD center [37].

Chronic rhinosinusitis is a multi-variant disease. Bacterial infection is the accepted cause of acute sinusitis in most circumstances, but the causes of chronic sinusitis are more complicated. *S. aureus *is a common pathogen that contributes to both acute and chronic rhinosinusitis [38]. The present study has added supportive data to the observation above. We identified *S. aureus *in the surgically removed tissues from the sinuses in 75% of the patients with UC-CRS and 100% of sinus samples showed positive mRNA for S. aureus. One of the features of CRS is purulent secretions into nasal cavity via the natural ostia. The nasal mucosa is covered with a lining of ciliated epithelium, covers with mucus blanket. One of the functions of this mucus blanket is the trapping of dust from inhaled air and secretions from the sinuses, and removing them from the nasal cavity by rhythmic locomotion. Because of the backward movement of the mucus blanket, some of the trapped substances such as SEB may be swallowed and enter the gastrointestinal tract to alter the mucosal physiological functions. The present data show high levels of SEB in the sinus wash fluids from the patients with UC-CRS. Previous reports also indicate high levels of SEB in nasal/sinus secretions of patients with chronic sinusitis, especially in those with polyposis [22]. The present data also showed nasal/sinus polyposis in 43.75% UC-CRS patients. Thus, it is quite possible that the SEB-containing secretions released from sinuses were swallowed and contribute to the pathophysiological changes in the colonic mucosa. The two groups of patients were treated with approximately the same remedies for their UC during the 12-week observation period. The different outcome between the two groups of patients that supports our hypotheses that CRS derived SEB plays a certain role in the pathogenesis of UC in these patients. However, the present data show that not all patients with *S. aureus*-infected CRS suffer from UC (Figure 9). Additional factors such as adjuvants may be required to act synergistically with SEB to induce intestinal disorders [27].

Serum IgE antibody level elevation is one of the main features of the type I allergic reactions including acute and chronic allergic diseases [39,40]. The accumulated evidence has emphasized that IgE mediated inflammation plays a role in the pathogenesis of ulcerative colitis [41,42]. We detected high levels of total serum IgE in the UC-CRS patients in this study. This phenomenon stresses that IgE mediated disorders are occurring in these patients. Further evidence we have obtained is that high levels of serum SEB specific IgE antibody were detected in the patients with UC-CRS. Although the antigen SEB that induced the specific IgE production in the present study could be from multiple sources, the fact that a significant reduction of serum SEB specific IgE after FESS indicates that the sinusitis derived SEB may be the obligate antigen. These SEB molecules are also capable of acting as superantigens and reacting with T lymphocytes, inducing massive activation, proliferation, and cytokine production by CD4+ T cells via specific Vbeta elements on the TCR, initiating inappropriate immune reactions in the local tissue and leading to IgE antibody production as well as inflammation [43].

We have located anti-SEB antibody positive stained cells in the colonic biopsies. The positively stained cells were identified in the patients with UC-CRS, but not in the normal colonic mucosa. The phenomenon indicates that there are anti-SEB antibody-bearing cells in the colonic mucosa of these patients. These cells can be the anti-SEB antibody secreting cells (such as plasma cells), or the cells (e.g., mast cells) have been bound by anti-SEB antibodies that are sourced from other cells. We may employ double-antibody staining technique with confocal microscopy in the future to identify the cell types. How have these anti-SEB antibody secreting cells been generated in the colonic mucosa? The mechanism of IgE production induced by SEB is also not clear. SEB from *S. aureus *is discharged to the nasal cavity from the sinuses, swallowed into the gastrointestinal tract, where the SEB is absorbed by the intestinal epithelial cells via a Toll like receptor (TLR) 2-mediated mechanism. Follicle cells in the Peyer's patch may be one of the paths to transport SEB from the luminal side to the lamina propria. TLR2 has been identified on the M cells of Peyer's patches in intestinal mucosa (44). SEB is one of the ligands for TLR2 (45). Once binding to TLR2 on the M cells, SEB may be internalized and transported across the thin cytoplasm of the M cells. SEB is then released by exocytosis from the basolateral membrane where the SEB can contact immune cells and induce inappropriate immune reactions, such as IgE production and inflammation. However, the detailed mechanism needs to be further understood. SEB then acts as a specific antigen and contacts immune cells in the lamina propria to initiate antibody production. Although this is the first study that reports anti-SEB positive immune cells in the colonic mucosa, there are some studies involved with SEB-colonic mucosa interaction have been published. Lu et al report that SEB compromises mouse colonic epithelial barrier function and induces inflammation in the mucosa [19,20]. McKay et al indicate that SEB induces colonic epithelial barrier deficiency via induction of IFN-γ and TNF-α production that can be partially inhibited by TGF-β [9,24,46]. Dionne et al observed that colonic biopsy specimens from UC patients produce TNF-α significantly more than normal controls in response to SEB stimulation [17]. Taken together, these findings suggest SEB plays an important role in the pathogenesis of chronic inflammatory intestinal disorders. The precise mechanism needs to be further investigated.

We have detected serum C reactive protein levels increasing in the patients with UC of both groups. The C reactive protein levels dropped significantly in the patients with UC-CRS after FESS. C reactive protein is an indicator of body inflammation and although it is non-specific, general elevation of serum C reactive protein in UC patients has been reported [47]. C reactive protein can be a biochemical marker that is useful to stratify patients likely to respond to biologic therapies and to follow response to treatment. The decrease of serum C reactive protein levels may be from the removal of inflammation in the sinuses [48] or from the amelioration of UC or both. Therefore, the decrease of serum C reactive protein can be a useful biological marker of removing inflammation from the sinuses and amelioration of the inflammation in colonic mucosa.

Mast cells play an important role in pathology and pathophysiology of UC [49]. The accumulated evidence shows activated mast cells in the colonic mucosa of the UC patients [49,50]. Our results are in line with previous reports. Furthermore, we provided quantitative data about mast cell activation by showing a significant difference in the ratio of degranulation in the mast cells of the patients with UC-CRS from the patients with UC only and the normal controls. Two types of mast cell degranulation, PMD and AND, were identified in the present study. PMD often presents in mast cells during chronic inflammation whereas AND is more likely in mast cells in acute situation such as in anaphylaxes [51]. In this study, electron microscopy revealed that even in the normal colonic mucosa there are a certain number of degranulated granules in the mast cells including both PMD and AND according to the criteria reported by Crivellato [52]. Most the degranulated granules in the mast cells from the UC patients were of the PMD type. This supports the definition that PMD type degranulation mainly attributes to chronic inflammation [51]. The incubation with SEB further increased mast cell degranulation in the colonic biopsy specimens with AND dominantly in the UC-CRS group. The mechanism of this phenomenon may be that the anti-SEB antibody belongs to the IgE class *per se*; anti-SEB IgE antibody bound to mast cells through the high affinity IgE receptors (FcåRI); SEB binds to the anti-SEB antibody-IgE receptor complex to activate mast cells.

Mast cells function as effector cells during immune inflammation by releasing chemical mediators. Histamine and tryptase are two of the main mediators and play important roles in both allergic inflammation and chronic inflammation [53,54]. The present study shows mast cell activation in colonic biopsies in response to SEB stimulation ex vivo by showing release of both tryptase and histamine, is consistent with previous reports [17]. The results implicate that when CRS-derived SEB arrives in the colon of these patients with UC-CRS, it will disturb intestinal mucosal function as described below. Tryptase is the main cytosol protein of mast cells, representing approximately 25% of the protein in mast cells [49]. A unique feature of tryptase is its ability to cleave and activate PAR2 receptors on several cell types including intestinal epithelial cells [53]. Intestinal epithelial barrier function may be compromised in response to PAR2 activation [55]. It may contribute partially (if not totally) to the deficiency of the intestinal barrier of patients with UC [56]. The fact that SEB induces tryptase and histamine release from the colonic biopsy specimens of the UC-CRS patients, but not from the UC-only patients or normal controls indicates that tryptase and histamine release is specific. One possible explanation for this is that the mast cells in the colonic mucosa have been primed by pre-binding with anti-SEB IgE antibody. The primed mast cells are then activated when they are re-exposed to SEB. Yet further investigation needs to be done to clarify the mechanism. Supporting evidence observed in the present study is that further increase in AND type degranulation in the mast cells of the colonic mucosa of the patients with UC-CRS after incubation with SEB implicates that the mast cells have had acute release of the granular contents within a short time in contrast of PMD type degranulation, the chronic release of the granular content [36,51]. The amount of tryptase and histamine released from the UC-only biopsies after incubation with SEB didn't show statistical difference from that before the addition of SEB that indicates only a spontaneous mediator released from these mast cells in the colonic tissue.

## Conclusion

We have reported a group of patients with both UC and CRS whose UC clinical symptoms were marked improved in addition to the amelioration of CRS after FESS. These data show a possible association between CRS and UC. There may be a subset of UC patients with high levels of IgE some of which is directed towards SEB. Although the present data are not sufficient to draw a conclusion on whether this is the initiating factor in UC or the progression factor, certainly it is a clue for us to further explore pathogenesis of UC about that we have had very limited knowledge yet.

## List of abbreviations

UC, ulcerative colitis; UCSS, UC severe score; CRS, chronic rhinosinusitis; SEB, Staphylacoccal enterotoxin B; FESS, functional endoscopic sinus surgery; EM, electron microscopy; PMD, piecemeal degranulation; AND, anaphylactic degranulation; HRP, horseradish peroxidase.

## Competing interests

The author(s) declare that they have no competing interests.

## Author's contributions

PY was involved in study design, a portion of FESS, histology, EM observation, data analysis and manuscript preparation; TL, BW and TZ were involved CRS treatment, FESS and some experiments; AY was involved in UC management; PZ was involved as a consultant in UC management; TD was involved as a consultant in sinusitis treatment.

## Pre-publication history

The pre-publication history for this paper can be accessed here:



## Figures and Tables

**Table 1:** Demographic and disease features of the patients before FESS

Group	Control	FESS
Sex	Male: 15; female: 17	Male: 18; female: 14
Age	33.23 (20 – 66)	35.82 (26 – 58)
Weight (kg)	62 (55 – 68)	60 (51 – 71)
Duration of UC (months)	28 (15 – 68)	35 (29 – 78)
Prednisolone equivalent (mg/day)	15 (5 – 20)	15 (5 – 20)

Data are shown as medians (interquartile ranges). UC, ulcerative colitis; FESS: functional endoscopic sinus surgery.
